# Spectral CT Imaging of Laryngeal and Hypopharyngeal Squamous Cell Carcinoma: Evaluation of Image Quality and Status of Lymph Nodes

**DOI:** 10.1371/journal.pone.0083492

**Published:** 2013-12-30

**Authors:** Aiyin Li, Hui Liang, Wei Li, Zhongzhou Wang, Tao Pang, Jun Li, Hao Shi, Chengqi Zhang

**Affiliations:** 1 Department of Radiology, Qianfoshan Hospital Affiliated to Shandong University, Jinan, Shandong Province, China; 2 Department of Otolaryngology, Qianfoshan Hospital Affiliated to Shandong University, Jinan, Shandong Province, China; Mayo Clinic College of Medicine, United States of America

## Abstract

**Purpose:**

The purpose of this study was to evaluate image quality and status of lymph nodes in laryngeal and hypopharyngeal squamous cell carcinoma (SCC) patients using spectral CT imaging.

**Materials and Methods:**

Thirty-eight patients with laryngeal and hypopharyngeal SCCs were scanned with spectral CT mode in venous phase. The conventional 140-kVp polychromatic images and one hundred and one sets of monochromatic images were generated ranging from 40 keV to 140 keV. The mean optimal keV was calculated on the monochromatic images. The image quality of the mean optimal keV monochromatic images and polychromatic images was compared with two different methods including a quantitative analysis method and a qualitative analysis method. The HU curve slope (λ_HU_) in the target lymph nodes and the primary lesion was calculated respectively. The ratio of λ_HU_ was studied between metastatic and non-metastatic lymph nodes group.

**Results:**

A total of 38 primary lesions were included. The mean optimal keV was obtained at 55±1.77 keV on the monochromatic images. The image quality evaluated by two different methods including a quantitative analysis method and a qualitative analysis method was obviously increased on monochromatic images than polychromatic images (p<0.05). The ratio of λ_HU_ between metastatic and non-metastatic lymph nodes was significantly different in the venous phase images (p<0.05).

**Conclusion:**

The monochromatic images obtained with spectral CT can be used to improve the image quality of laryngeal and hypopharyngeal SCC and the N-staging accuracy. The quantitative ratio of λ_HU_ may be helpful for differentiating between metastatic and non-metastatic cervical lymph nodes.

## Introduction

Laryngeal and hypopharyngeal SCC are one of the most common malignant tumors in head and neck tumor. The extent of the tumor and the detection of metastatic lymph nodes have essential impact on treatment decision for laryngeal and hypopharyngeal SCC, and tumor volume measured by pretreatment CT is increasingly considered to have the highest prognostic impact regarding local recurrence[1]–[7]. Tumor volume would be able to accurately measure depends on the better image quality. But the extension and volume of laryngeal and hypopharyngeal SCC can not accurately be assessed by conventional CT imaging examination [8]. Sometimes, because of the tumor is too small to find, the best treatment opportunity is missed. How to improve the CT image quality is always a problem. The presence of metastatic lymph node means a significantly poor prognosis of the disease. Two most frequently used imaging methods, contrast-enhanced computed tomography (CT) and magnetic resonance imaging (MRI) allow detection of enlarged nodes with necrosis and external diffusion [9], but neither method can accurately differentiate non-metastatic from metastatic, non-enlarged lymph nodes [10].

With the emergence of spectrum CT, those are possible to be solved. The spectral CT has higher soft tissue resolution and contrast-to-noise ratio (CNR) than conventional multislice CT. The spectral CT was capable of extracting quantitative information about the elemental and molecular composition of tissue and contrast materials basing on their attenuation properties [11]. The goal of the study was to evaluate the usefulness of spectral CT for image quality improvement and the differentiation between metastatic and non-metastatic cervical lymph nodes in patients with laryngeal and hypopharyngeal SCC.

## Materials and Methods

### Patients

The study was approved by Qian fo shan Hospital Ethics Committee. Written informed consent was obtained from each patient before imaging. We reviewed all consecutive patients who underwent surgery for resectable SCC and target lymph nodes in laryngeal and hypopharyngeal between June 2010 and December 2012 in Qian fo shan Hospital. The patients with other tumors were excluded from the study group. The diagnoses of all the patients were confirmed by the post-operative pathological examination (including the primary lesion and target lymph nodes).The 38 laryngeal and hypopharyngeal SCCs that were operated on within ten days (ranging from 3 to 10 days) after CT scans were enrolled in this study. This study included 24 men and 14 women. Their mean age (SD) was 61.13 (8.44) years old. There were 12 patients with glottic cancer, 8 with supraglottic cancer, and 18 with hypopharyngeal cancer. There were 72 lymph nodes were included totally, which consist of 30 metastatic and 42 non-metastatic lymph nodes. The clinical data and demographics were given in **Table 1**. The surgeon (H Liang) studied the CT images carefully to map the lymph nodes, and marked them intraoperatively according to the findings on CT images. Locations of lymph nodes were recorded. To improve the certainty that the lymph nodes seen on CT were accurately correlated with the lymph nodes on surgery, only the lymph nodes larger than 6 mm were included in this study [12].

**Table 1:** Patient Characteristics and Scanning Parameters.

	metastatic lymph nodes	non-metastatic lymph nodes		*P* Value
**Age (y)** †	62.53(7.93)	61.5(7.63)	*t = *0.557	0.579
**Male to female ratio**	17/13	29/13	?2 = 1.163	0.281
**Primary site**			?2 = 2.712	0.258
**Supraglottic** ‡	8	14		
**Glottis** ‡	3	9		
**Hypopharyngeal** ‡	19	19		
**Tube voltage**	140 kv/80 kv	140 kv/80 kv		NA
**Tube current**	Less than 640 mAs	Less than 640 mAs		NA
**Rotation time**	0.6 s/rot	0.6 s/rot		NA
**Pitch**	0.531	0.531		NA
**Collimation**	20 mm	20 mm		NA
**Scan slice thickness**	0.625 mm	0.625 mm		NA
**Reconstruction slice thickness**	0.625 mm	0.625 mm		NA

NA _ Not applicable.

Data are means _ standard deviations.

Data are numbers of lymph nodes.

### Image acquisition

After abrosia for 4 hours, CT scan was performed with a high-definition CT scanner (Discovery CT750HD, GE Healthcare). The unenhanced and arterial phases used standard MDCT. The venous phase (70S) contrast-enhanced scan was performed using the dual energy spectral CT mode with a single tube, fast kilo-voltage switching between 80 kVp and 140 kVp in less than 0.5 msec. CT scans were obtained through the area from the hyoid bone to the bottom of the cricoid cartilage, with 0.625-mm axial images. Patients were injected with contrast media (iopromide, Ultravisr300; Schering, Berlin, Germany) by using a power injector at a rate of 3 ml/s a total of 85–100 ml (1.5 ml per kilogram of body weight) was injected intravenously.

### Evaluation of primary lesion image

#### Quantitative analysis

A head and neck tumor radiologist (Z.W), with 5 years' experience in CT diagnosis, performed all the measurements to review the polychromatic images and the mean optimal monochromatic images with a same soft-tissue display window preset (WL 120 and WW 240). Images were reconstructed with a 20 cm display field-of-view, 512×512 reconstruction matrix size. Polychromatic images were corresponded to the conventional imaging, and monochromatic images sets corresponded to photon energies ranging from 40 to 140 keV, and the monochromatic images were sent to a special gemstone spectral imaging (GSI) viewer for analysis.

**Figure 1:**
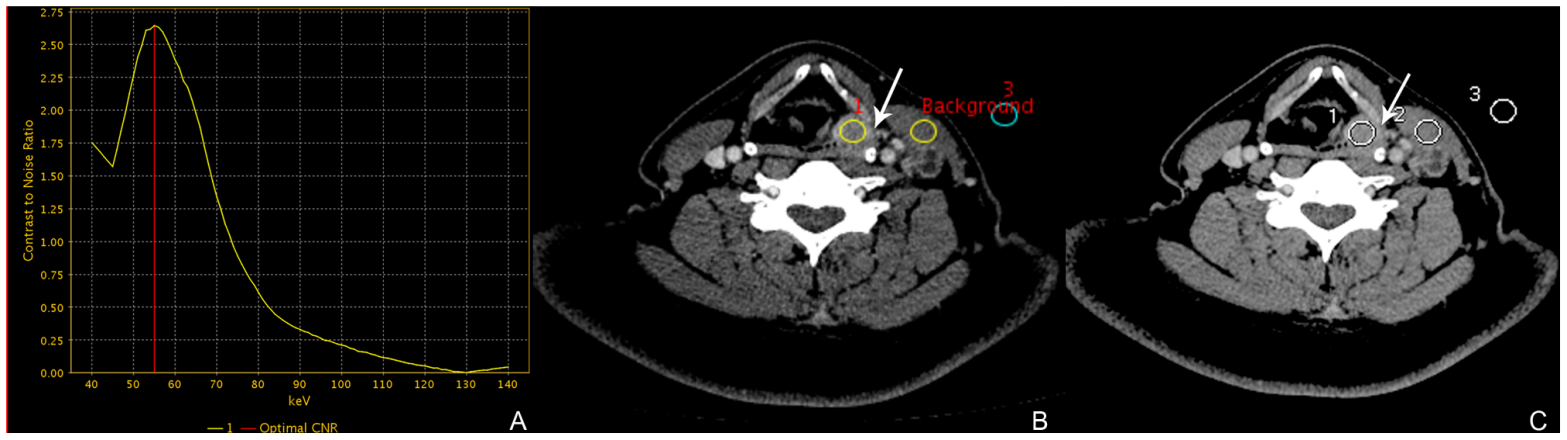
Enhanced axial images in venous phase from a 58-year-old patient with supraglottic SCC, for objective evaluation of image quality. A, B and C are the optimal CNR curve, 55(A) Demonstrating the GSI Viewer calculation of the CNR curve, optimal monochromatic energy of 55 keV achieved the best CNR for the primary lesion, and the locations of the background ipsilateral sternocleidomastoideus (RIO_background_) and primary lesion (RIO_1_) to generate the curve on (B). The air region (RIO_3_) is evaluated as the index of image noise. (C) The size, shape and position of the ROIs are kept consistent with image (B), and the primary lesion is less visible than that on the monochromatic image at 55 keV (arrow). On image (B), contrast: 32.16, noise: 7.83, CNR: 4.11; on image (C), contrast: 16.23, noise: 7.02, CNR: 2.31.

From the monochromatic image sets, the GSI Viewer software package automatically calculated and displayed the CNR values for the 101 sets of monochromatic images real time. From the CNR plot, the optimal single energy (keV) level of 38 lesions for generating the best CNR between the primary lesion and the ipsilateral sternocleidomastoideus of the same slice could be selected (**Fig 1A**). The mean optimal keV was calculated. Single maximal axial tumor diameter was recorded for the monochromatic images with the mean optimal keV.

The mean CT data of primary lesions were assessed by manually placing circular or ovoid ROIs, which were drawn to encompass the hyperenhancing portion of the lesion as possible. The mean CT data as the contrast regions were obtained by manually placing circular or ovoid ROIs in the ipsilateral sternocleidomastoideus of the same slice. The image noise was based on the standard deviation of the pixel values within surrounding air in front of the patient. The size, shape and position of the ROIs were kept consistent between the polychromatic images and the monochromatic images (**Fig. 1B, C**). All RIOs we measured were far away from the bones as possible to improve the accuracy. To ensure consistency, all measurements were repeated three times and average values were calculated.

The contrast-to-noise ratio (CNR) of the polychromatic images and the mean optimal keV monochromatic images were calculated by using the following equation: CNR = (ROI_lesion_ – ROI_muscle_)/SD_air_ [13], where ROI_lesion_ and ROI_muscle_ are the mean attenuation of the primary lesion and the ipsilateral sternocleidomastoideus of the same slice respectively, and SD_air_ is the standard deviation of the pixel values within surrounding air in front of the patient. The CNRs were determined for the venous phases.

#### Qualitative analysis

The qualitative analysis of CT images obtained in the polychromatic images and the mean optimal keV monochromatic images at ADW 4.4 workstation were assessed by two radiologists (T.P, 20 years of experience in head and neck tumor CT; A.L, 13 years of experience) in consensus.

For image quality, the scale was as follows: score of 5 = no obvious image noise and artifacts, sharp structure of lesion and satisfactory details; score of 4 =  mild image noise and artifacts, less clear structure of lesion and details; score of 3 =  moderate image noise and artifacts, decreased confidence in details but structure of lesion still relatively clear; score of 2 =  severe image noise and artifacts, confidence in details and structure of lesion decreased; and score of 1 =  severe image noise and artifacts, non-diagnostic[14].

### Evaluation of cervical lymph nodes

All lymph nodes were analyzed by application of the monochromatic images. ROIs were placed on primary lesions and target lymph nodes. All RIOs were also far away from the bones as possible to improve the accuracy. For each measurement, a total of 3 ROIs were applied and average value was calculated. For homogenous nodes, ROIs could be drawn to cover hyperenhancing portion of the lymph region. For inhomogeneous nodes, ROIs were placed on the nodes corresponding to solid components. For primary lesions, ROIs were placed on hyperenhancing portion of the lesion as possible. Two radiologists (H.S and T.P) blinded to the diagnosis drew the ROIs independently, and in questionable cases, a radiologist with 21 years' experience (C.Z) was consulted to reach consensus. Quantitative HU curve slope (λ_HU_) of primary lesion and target lymph node was obtained as the CT attenuation difference at 2 energy levels (40 and 100 keV) divided by the energy difference (60 keV) from the λ_HU_ = (CT_40keV_-CT_100keV_)/60[15]. The ratio of λ_Hu_(λ_Huln_/λ_Hulesion_, λ_Huln_ and λ_Hulesion_ are the λ_HU_ of target lymph node and the primary lesion respectively) was calculated in every patient.

### Statistical analysis

All images data were analyzed by using dedicated statistical software (SPSS for Windows, version 16). A value of P<0.05 was considered statistically significant. The mean optimal keV, age, CT value difference between the primary lesion and the sternocleidomastoideus, image noise values, contrast-to-noise ratios, subjective image quality and the ratio of λ_Hu_ were presented as mean ± standard deviation (SD). Paired t-test was performed on the contrasts, image noise values and contrast-to-noise ratios for the polychromatic and the mean optimal keV monochromatic image sets. Paired t-test was performed on the subjective scores with the statistical significance of p<0.05.

The ratio of λ_Hu_ in the metastatic and non-metastatic lymph nodes was analyzed by using independent t-test. Characteristics (age, sex and the location of primary lesion) of both patient groups (metastatic lymph nodes and non-metastatic lymph nodes) were compared by using the independent *t* test and χ^2^ test respectively, and pathological classification of both patient groups was analyzed by using ANOVA test. Pearson correlation coefficient was calculated for both radiologists, and R≥0.8 was considered to have good correlation.

## Result

### Evaluation of primary lesion image

#### Quantitative analysis (Table 2)

**Table 2:** Contrast, CNR and image noise for the laryngeal and hypopharyngeal SCC with the monochromatic 55

	laryngeal and hypopharyngeal SCC		
	Contrast	Noise	CNR
55 kev Monochromatic image	61.22 (13.81)	8.78 (0.93)	7.03(1.63)
Polychromatic image	31.03 (7.01)	7.27(0.49)	4.31(1.10)
*t* -Value	21.83	12.62	17.73
*P*-Value	.000	.000	.000

Numbers in parentheses are standard deviations. Contrast: CT value difference between the primary lesion and the sternocleidomastoideus; Noise: the standard deviation of the pixel values from the air region; CNR: contrast-to-noise ratio.

Thirty-eight laryngeal and hypopharyngeal SCCs had a mean tumor diameter (SD) of 3.14±1.46 cm. The mean optimal keV for displaying laryngeal and hypopharyngeal SCC in our patient population was 55±1.77 keV. The 55 keV for viewing primary lesion was selected. We measured the CT value difference, image noise values and CNR for the primary lesion differed on the basis of 55 keV monochromatic and polychromatic images. The CT value difference and CNR value were 61.22±13.81 and 7.03±1.63 at 55 keV respectively, which were statistically higher than 31.03 (7.01) and 4.31(1.10) from polychromatic images (p<0.05). The mean image noise value on monochromatic image was higher than that from polychromatic image (8.78±0.93 vs. 7.27±0.49, p<0.05).

#### Qualitative analysis (Table 3)

**Table 3:** Subjective image scores.

	Image quality score
55 kev Monochromatic image	4.58(0.50)
Polychromatic image	3.66(0.53)
*t* -Value	11.67
*P*-Value	.000

Numbers in parentheses are standard deviations. A five-point ordinal scale (see “Qualitative analysis”) was used. P<0.05 indicates a statistically significant difference between group polychromatic and Monochromatic (55 kev) images.

**Figure 2:**
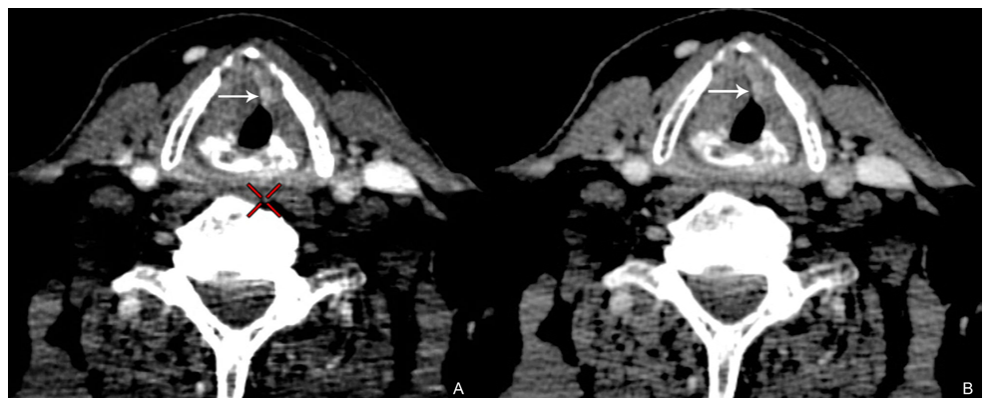
Transverse (A) monochromatic image at 55-keV energy level and (B) polychromatic images with the same window width/level obtained from spectral CT acquisition (section thickness, 0.625 mm) in 57-year-old man with glottic laryngeal SCC. The edge of the primary lesion is more clearly displayed and the image contrast is better on 55-keV monochromatic image than the polychromatic image with acceptable image noise through visual observation (arrow).

According to the reference standard, image quality of 55 keV monochromatic and polychromatic image was evaluated. Significant statistical difference was noted between subjective scores in the two image sets. The subjective image score for the 55 keV monochromatic images was higher (better) than the polychromatic images (4.58±0.50 vs. 3.66±0.53, p<0.01). The edge of the primary lesion was more clearly displayed and the image contrast was better on 55 keV monochromatic image than the polychromatic image with acceptable image noise through visual observation (Figure 2).

### Evaluation of metastatic lymph nodes

**Figure 3:**
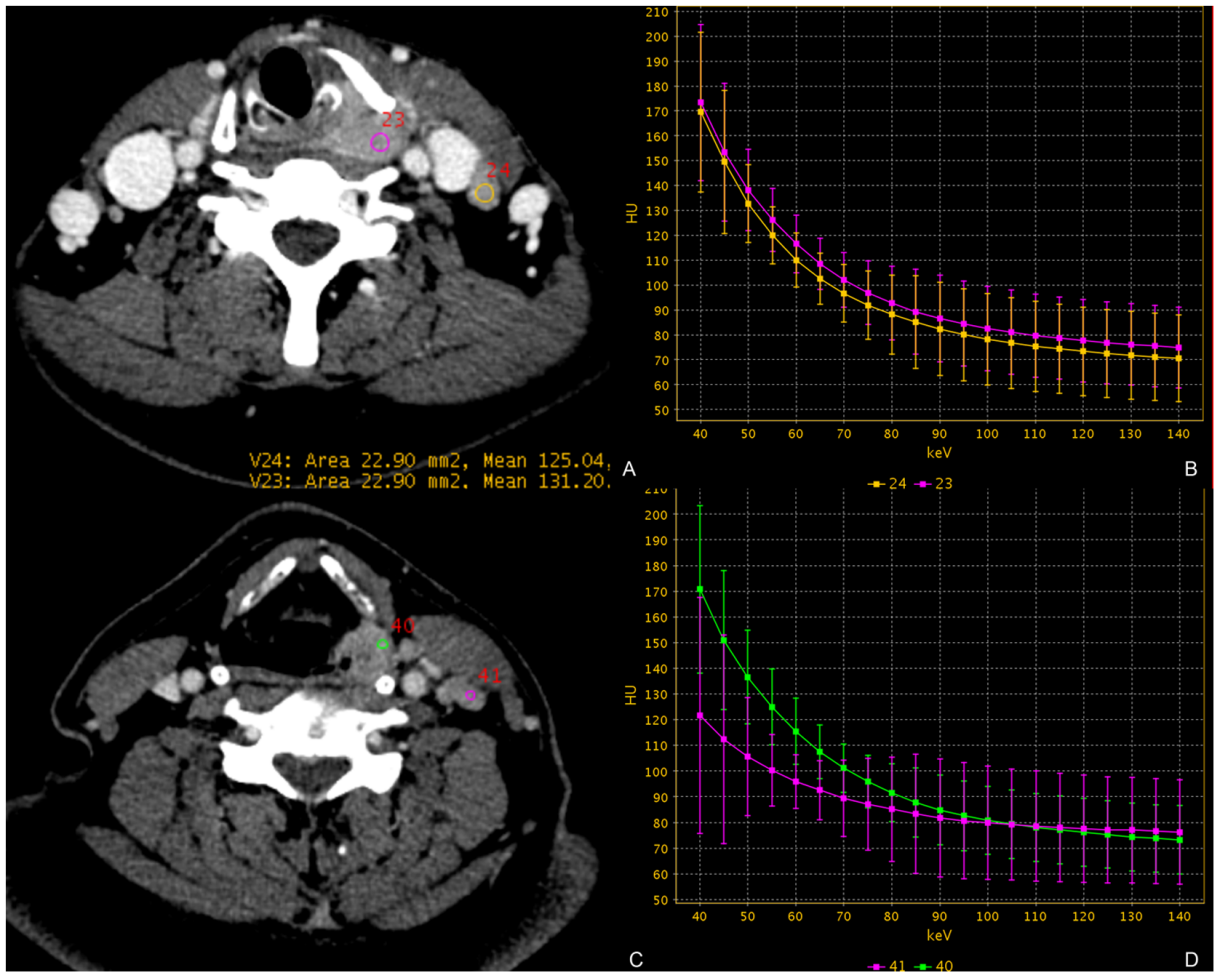
Lymph nodes are evaluated in 69-year-old man with laryngeal SCC. (A and C) Demonstrates the region of interest in primary lesion and target lymph nodes (region 23, 40 =  primary lesion; region 24 =  metastatic lymph node; region 41 =  non-metastatic lymph node) on a monochromatic image. (B) Demonstrates the spectral HU curves (CT values in Y axis vs. keV in X axis) of primary lesion (pink line) and metastatic lymph node (yellow line). The λ_Hu_ of primary lesion: 1.5, the λ_Hu_ of metastatic lymph node: 1.53, the ratio of λ_Hu_: 1.02. (D) Demonstrate the spectral HU curves (CT values in Y axis vs. keV in X axis) of primary lesion (green line) and non-metastatic lymph node (pink line). The λ_Hu_ of primary lesion: 1.5, the λ_Hu_ of non-metastatic lymph node: 0.7, the ratio of λ_Hu_: 0.47.

**Figure 4:**
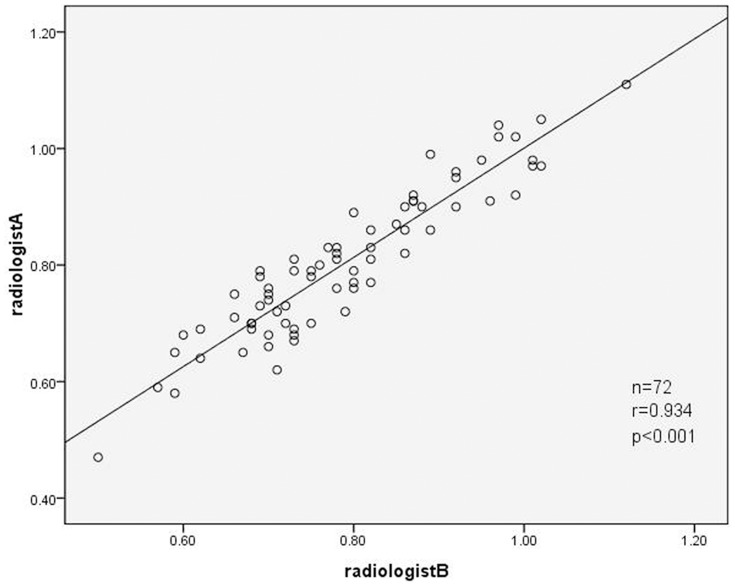
Correlation plot of the ratio of λ_Hu_ between radiologist A and radiologist B in lymph nodes. There is a significant strong correlation in the ratio of λ_Hu_ between radiologist A and radiologist B (*r* = 0.934). n: number of lymph nodes, *r*: pearson correlation coefficient.

Comparison of the two patient groups (metastatic lymph nodes and non-metastatic lymph nodes) did not reveal any significant differences in patient age, sex and primary site (**Table 1**).The ratios of λ_Hu_ in the metastatic lymph nodes were 0.92±0.09 and 0.90±0.10 by two radiologists, and the ratios of λ_HU_ in the non-metastatic lymph nodes were 0.72±0.08 and 0.71±0.08 respectively. The ratio of λ_HU_ between metastatic and non-metastatic lymph nodes was significantly different (p<0.05, **Table 4,** **Fig 3**). There was no significant difference between the two radiologists, with good correlation (r = 0.934, p<0.001, **Fig 4**). The ratio of λ_HU_ was not related to pathological classification (p>0.05).

**Table 4:** Evaluation of lymph node.

	Ratio of λ_Hu_	
	A	B
non-metastatic lymph nodes	0.72(0.08)	0.71(0.08)
metastatic lymph nodes	0.92(0.09)	0.90(0.10)
*t* -Value	−9.864	−8,744
*P*-Value	.000	.000

λ_Hu_ = (CT_40keV_-CT_100keV_)/60; Ratio of λ_Hu_: λ_Huln_/λ_Hulesion_(λ_Huln_ and λ_Hulesion_ is the λ_Hu_ of target lymph node and the primary lesion respectively); Numbers in parentheses are standard deviations. A and B represented the two radiologists.

## Discussion

The therapeutic decision in cases of laryngeal and hypopharyngeal SCC is based on the tumor stage and grading as well as on the patient's general conditions. The tumor stage is traditionally defined by TNM classification, according to site, local extension of the disease, mobility of the vocal apparatus, and lymph node status. In clinical practice, several studies reported that the volume of the neoplasm is stronger predictive factors of local recurrence [1], [3]–[7]. However, the introduction of multisection CT has resulted in little progress in image interpretation [16], and tumor volume is still overestimated by CT and MR imaging, resulting in unnecessary total laryngectomies in some patients [17]. Sometimes, it is due to the technical difficulty of distinguishing the boundary of these tumors.

Spectral imaging obtained with the single tube, rapid dual tube voltage switching technique provides the monochromatic images depicting how the imaged object would look if the X-ray source produced only single energy X-ray photons. This would allow for increased image contrast resolution, and it has been proved by some reports [18].

Our study results demonstrate that the images quality. The lesion detectability and the conspicuity of laryngeal and hypopharyngeal SCC can be improved by selecting the optimal mean energy level (55 keV) of monochromatic imaging compared with the polychromatic imaging. We observed a percentage increase in CNR by 63% for laryngeal and hypopharyngeal SCC at the 55 keV image than polychromatic image. There was significant statistical difference between the 55 keV and polychromatic image. Even though the image noise was about 21% higher at 55 keV, but the qualitative subjective score regarding primary lesion image quality was much higher than the polychromatic image. In general, the image quality of primary lesion in the 55 keV image was significantly improved than in the polychromatic image. The fact is that the spectral CT scan mode allows the radiologists to choose an optimal imaging mode to accurately evaluate the boundary of tumor invasion and calculated the tumor volume exactly. It provided the accurate prognostic information for predicting local tumor control.

The detection of cervical lymph node metastases in laryngeal and hypopharyngeal SCC entails a significantly poorer prognosis of the disease and increases the rate of morbidity, mortality, recurrences and complications after treatment. There is consensus among most authors that the appearance of lymph node metastasis decreases the chance of survival by approximately 50% [19]. Therefore, the detection of cervical lymph nodes in laryngeal and hypopharyngeal SCC patients is essential.

At present, radiologists commonly use techniques include CT scan, MRI scan, PET/CT, and so on [20]–[22]. The standard used by CT and MRI in the staging of lymph nodes metastases of laryngeal and hypopharyngeal SCC is the size of the lymph nodes, irregular edges of the nodes, enhancement of the nodes, etc. The studies mentions different radiological criteria that have been used in the evaluation of neck lymph nodes metastases. Mancuso et al. [23] considered as metastases those lymph nodes over 15 mm, while Som et al. [24] considered the lower limits of the cervical lymph nodes, to be accepted as metastases, are 10 mm. Other author considered the ability of CT to detect metastatic lymph nodes in head and neck tumors is quite acceptable, but, it is less so for correctly staging them [25]. PET/CT exam has its own limitations because scanner resolution also limits the evaluation of small malignancies or sub-centimeter nodal metastases which may not be detected [26]. Therefore, it is necessary to look for other imaging means that provide greater accuracy of N staging to avoid unnecessary elective neck dissections.

Spectral CT image could be helpful to improve the accuracy of N staging. In our study, we retrospectively analyzed 38 primary laryngeal and hypopharyngeal SCCs and 72 lymph nodes were consisted of 30 metastatic and 42 non-metastatic lymph nodes. The ratios of λ_Hu_ in the metastatic lymph nodes group were 0.89±0.19 and 0.90±0.10. The ratios of λ_Hu_ in the non-metastatic lymph nodes group were 0.72±0.12 and 0.71±0.78 respectively. Statistical analysis showed a significant difference between metastatic lymph nodes and non-metastatic lymph nodes groups in the ratio of λ_HU_ (p<0.05). Spectral CT is sensitive for detecting lymph node metastases due to its ability to detect microscopic lymph nodal invasion. A substance behaves at two different energies can provide information about tissue composition beyond that obtainable with single-energy techniques [27]. Spectral CT is able to extract quantitative information about the tissue composition and contrast materials basing on their attenuation properties. Compared with the primary lesion, metastatic lymph nodes could be with similar tissue composition and contrast materials information than non-metastatic lymph nodes. The results show that the ratio of λ_HU_ in lymph nodes can be used as a quantitative parameter to distinguish benign lymph nodes from malignant lymph nodes.

This study does have several limitations. First, spectral CT is an imaging method that extends the capabilities of conventional CT. It is noticed that the monochromatic images were “calculated” rather than from the true monochromatic. Second, this study only compared image quality between monochromatic and polychromatic images. The volume of the primary lesion was not measured, and it was not considerate to outline tumor margins because of the complex spreading process and the presence of inflammation, edema and necrosis. Further research needs to be performed to provide more powerful evidence for prognosis of the tumor. Third, there is variability in the transit time from IV injection to systemic circulation leading to variability in the absolute values of λ_HU_. Therefore, we used the ratio of λ_HU_ (λ_Huln_/λ_Hulesion_) instead of absolute values to account for this because the transit time to lymph node should approximately equal the transit time to the lesion in question. This method can minimize the error, but it does not absolutely avoid the error. Forth, the low-energy monochromatic imaging (55 keV in this case) is more prone to beam-hardening artifact than high-energy monochromatic images. Therefore, the RIOs we measured were far away from the bones as possible to improve the accuracy. Because of the location of laryngeal and hypopharyngeal tumors, the artifact from bone cannot be completely eliminated in the current study. We will try to use a vendor-specific metal artifact reduction software (MARS) in our future research to reduce the possible variability caused by beam hardening artifacts. Finally, the analysis of lymph nodes may increase more workload for the radiologist doctor. Patient's CT images were likely to display a lot of lymph nodes. To achieve a more accurate result of the lymph nodes, the radiologist must have a good acknowledgement of the patient's clinical condition and have to choose many lymph nodes in the imaging. This would be a heavy work for imaging post-processing.

In conclusion, spectral CT imaging provides both the monochromatic images and polychromatic images. The monochromatic images may be used to improve image quality on optimal mean energy images (55 keV) and the N staging accuracy for laryngeal and hypopharyngeal SCC. The quantitative measurement of ratio of λ_Hu_ on monochromatic images may be helpful for differentiating metastatic and non-metastatic lymph nodes. Spectral CT may provide new opportunities for detailed preoperative evaluation of not only the laryngeal and hypopharyngeal SCC morphology but also the N staging.
